# ED Stories: An Online Emergency Medicine Residency Community Building and Wellness Initiative

**DOI:** 10.7759/cureus.5405

**Published:** 2019-08-16

**Authors:** Geoffrey B Comp, Meenal Sharkey

**Affiliations:** 1 Emergency Medicine, Maricopa Medical Center, Phoenix, USA; 2 Emergency Medicine, OhioHealth Doctors Hospital, Columbus, USA

**Keywords:** residency education, wellness, group discussion, burnout, residency, slack, innovations in emergency medicine, medical education

## Abstract

The “ED Stories” project is a novel method for promoting individual wellness and interpersonal relationships on a virtual platform in an emergency medicine residency program. We engaged multiple individuals across all levels of medical training on a virtual platform to facilitate discussion regarding difficult situations, patient interactions, and tips on how to succeed in challenge circumstances in the future. The platform was outlined as a “safe space,” free of judgment and criticism. We engaged 54 participants with a culmination of 131 interactions; there were 14 original posts with a total of 22 comments. We believe that this virtual platform allowed for safe discussion of difficult concepts related to our jobs and served as an outlet to promote physician wellness and reduce burnout in the future.

## Introduction

Physician wellness is a complex issue that has recently become a focus in the medical education community. Many studies have indicated an alarming rate of burnout and high levels of stress in residents and medical students, with higher rates in emergency medicine when compared with other specialties [1-5]. Burnout is a state of mental fatigue characterized by emotional exhaustion, depersonalization or cynicism, as well as a sense of reduced personal accomplishment [1,5-6]. The syndrome has been linked with a variety of negative effects including provider depression, suicide, illicit drug use, and risk of medical errors [1-3].

Various methods have been designed and implemented to measure the characteristics and symptoms of burnout and well-being in subjects within different levels of medical training [6-7]. Strategies for implementing wellness programs have been proposed that focus on topics such as art therapy, counseling interventions, mindfulness training, communication skill training, breathing exercises, and organized leadership education [8-12]. Research has demonstrated significant improvement in measured signs of burnout after providing targeted education or interventions to help identify these symptoms early on before a negative event occurs [13-16].

The sharing of meaningful experiences and stories with peers may improve interpersonal relationships and help strengthen the residency community. In addition, “unloading” some of the more difficult cases may help prevent internalization and demoralization. Previous efforts with similar goals have focused on live communication events [17-19]. However, there is an opportunity for the discussion to be held on a virtual platform. We performed a limited PubMed and Google Scholar search for English articles using keywords including "intervention," "discussion forum," or "support group," with parameters to control for "virtual," or "asynchronous," and found limited results with no publications discussing this approach for emergency medicine residents. 

## Materials and methods

This initiative was developed to meet the following objectives: assess if an opportunity for discussion on a virtual platform would be utilized by invited participants; serve as an outlet for “difficult to discuss” cases to help an individual process and share experiences; provide a virtual safe space and guidance for the EM residency members to share stories and experiences; and promote bonding between residents and attending physicians with the goal of strengthening the educational community.

The project began with a brief lecture and introduction to the “ED Stories” project, as well as an explanation of program goals and objectives. A unique page was preemptively created and hosted on the Slack digital-messaging platform (Slack Technologies) used by the residency, a community-based four-year program with eight residents per year, for general program communication. One senior resident and one core faculty member were selected to act as moderators. All emergency medicine residents and faculty were encouraged to voluntarily join the specific conversation site. Participation was not required. They were invited to use this space to write about their thoughts and challenges, as well as professional and personal stresses. Other participants then had the opportunity to read the post and directly comment, or select other “reactions,” small ideograms linked to the post. Participation was voluntary and included writing posts as well interacting with previous messages. The “ED Stories” page listed specific goals of the project and initial examples of posts. 

## Results

The program was initiated in November 2018. Fifty-four people were invited to participate. In the first month of the implementation of the “ED Stories” project, 81 interactions were recorded on the page. There were 10 unique written posts, 17 comments, and 54 other reactions. Seventeen people (11 residents, five attending physicians, and the program coordinator) posted unique messages (Table 1). The program is still an active resource with continued participation. From November 2018 to March 2019, there has been a total of 131 recorded interactions, 14 unique posts, 22 comments, and 95 other reactions (Table 2).

**Table 1:** Initial first month participation in ED Stories Program

Participation	
Invited	54
Active	17
Resident	11 (65%)
Attending	5 (29%)
Other (program coordinator)	1 (6%)
Page Interactions	
Total	81
Unique Posts	10 (12%)
Comments	17 (21%)
Other Reactions	54 (67%)

**Table 2:** November 2018 to March 2019 participation in ED Stories Program APP, advanced practice provider

Participation	
Invited	54
Active	25
Resident	15 (60%)
Attending	8 (32%)
Other (program coordinator/APP)	2 (8%)
Page Interactions	
Total	131
Unique Posts	14 (11%)
Comments	22 (17%)
Other Reactions	95 (73%)

## Discussion

The initial goal of this program was to assess if an opportunity for discussion on a virtual platform would be utilized by the participants and provide potential space for community building and sharing. Others have reported benefit from being an active observer without personal submission through verbal feedback to the coordinators. While there was a decrease in specific participation in the last few months of the program, there is still continued activity. Additional participation by moderators including posing difficult scenario situations for people to discuss to stimulate conversation would be one way to maintain momentum and engage the participants. The potential for future expansion is to invite other ED staff members, including nursing staff, advanced practice providers, and even emergency medical services (EMS) support to facilitate discussion and add additional perspectives.

As this is a brief observed pilot evaluation to review utilization, participants were not surveyed on the personal benefit of the program. This is a significant limitation in evaluating to see if specific wellness objectives are met and addressed. However, future initiatives can focus on directly surveying the participants to obtain feedback regarding the program, and if they feel that they are benefiting from the discussions. This would allow for additional changes to the program to maximize participant benefit. Another limitation of the program is the self-selection of participants. As there are varying levels of comfort with sharing in a public forum, residents may feel reluctant to share vulnerable thoughts and feelings. Unfortunately, anonymous posting is not supported by the Slack channel. However, the moderators could make themselves available to post for an individual not comfortable with publicly sharing their experience. 

Resident support groups and debriefing sessions have been used as a resource for sharing professional struggles and clinical experiences, eliciting support, establishing relationships, and providing a forum for discussion [17-18]. One study of residents early on in training showed sessions aimed to reduce the prevalence of burnout were well received with 90% of the participants indicating that the intervention was a source of emotional and social support [19]. 

The impact of social networks to influence both positive and negative emotions and behaviors demonstrated by an individual has been previously reported [20]. Elevated levels of loneliness can result in a higher incidence of personal and work-related burnout. Residents with a higher number of social connections and a strong sense of an established location and role within their social network appear to have lower levels of loneliness [21]. Many residents identify peer relationships as one of the most critical sources of support throughout their medical training [17].

However, barriers to use of these types of community-building sessions, both real and perceived, including confidentiality, accessibility, and time commitment may inhibit participation [10,22]. The identification and management of these concerns are paramount to the success of wellness programs. An additional limitation can be a lack of time to reflect on a stressful situation. Sometimes, details of a specific event may be forgotten if a large amount of time passes. By providing an outlet to address these feelings in real time, they may describe the events more accurately and grow from the experience. The ED stories project aimed to provide an alternative solution to some of these difficulties while providing additional space for these conversations.

## Conclusions

The use of this virtual space appears to provide a useful area for members of the emergency medicine residency to post and react to difficult and sometimes highly emotional situations at their convenience. There are future plans to assess the effectiveness of this initiative and encourage additional use of the space. Other programs could easily implement a similar system using their specific platform for communication. We hope to inspire other residency programs to create similar projects that foster an environment of shared experiences and emotions that can empower an individual and strengthen the community.
